# Large Doses of Calomel in True Croup

**Published:** 1884-08-20

**Authors:** 


### LARGE DOSES OF CALOMEL IN TRUE CROUP.

Dr. O. T. Schultz contributes a very interesting paper on this subject to the American Practitioner for May, 1884. He first details the method introduced and extolled by Dr. Reiter, of Pittsburgh, Pa., who holds that this inflammation is due to "too much fibrin in the blood," which condition is produced by the liver having lost its fibrin-destroying power, and that mercury is the specific for compelling the liver to resume this function. Be his theory right or wrong, Dr. Reiter has put it into practice with great boldness, and with astonishing results. And he anchors his faith fully and squarely upon mercury in all forms of pseudo-membranous inflammation — fibrinous, septic and gangrenous, sthenic and asthenic—and without ever engaging in those delicate subterfuges, steaming, burning or cutting. His results are reported to be marvelous, and unattended with any unpleasant after effects, while the boldness with which he pushes mercury makes one's hair stand on end. Dr. Schultz then goes on to relate the case of his own boy, whom he treated according to Dr. R.'s method. He says:

After all the usual remedies had been tried and failed, in my despair I concluded to try Reiter's method. The treatment was begun at 9 a. m. on the 13th. The patient was given calomel: twenty grains at 9 a. m., ten grains at 10, five grains each at 11. 12, 1, 2:30, 4:30, 6:30, 8:30, 10:30, 12:30, 2:30, and 5 a. m. of 14th, being *eighty-five grains* in twenty-four hours. No other remedial measure was made use of.

"During this time his condition was as follows: At 12 m. of the 13th he is sleeping quietly, breathing easily; the hard sound in inspiration and expiration is replaced by a soft gurgling or bubbling; the cough is loose; large, loose rales are heard in the larynx; the surface is warmer than normal, soft but not moist; the pulse is very fast and small. At 2 p. m. profuse vomiting; glairy mucus with yellowish flakes; there is one thin, loose, not fetid passage. At 2:30 no more dyspnœa; stridor entirely absent; voice clearer; stronger; cough not frequent, loose, barking. Vomited again at 2:45; water and flakes looking like membrane, bearing occasional clots of blood; a similar vomit at 4, and a thin, loose stool containing flakes of white mucus. At 6 is bright, playful, breathing noiselessly; eats some, which he has not done since yesterday; cough still hoarse, loose; voice clear, possessing some timbre, not much given to creaking. Went to sleep at eight o'clock, sleeping quietly, breathing normally, without any rattling in larynx or any signs of dyspnœa; no fever; an abundant, dark passage, coming on rather hurriedly at 11. From this on he slept soundly and well till morning, his sleep broken only by occasional barking cough and by taking his medicine. At 7 a. m. of 14th he had another dark passage; the voice is clear; cough—at times hoarse, at times not; loose; respiration easy and noiseless; some feverishness, and pulse is rapid and small. Hydrargyrum bichlorid., one-sixteenth grain, is ordered every two hours; but the second dose causing vomiting in half an hour, the dose is reduced to one thirty-second of a grain. About noon began to complain of frequent attacks of belly-ache. Cough is only at times hoarse; respiration is easy; voice is clear; no appetite; no fever. By 4 p. m. has had two passages; the pain in the abdomen continues—is paroxysmal. In the evening the cough appears drier and is more frequent, and has the characteristics of bronchial cough; small dry rales are heard now for the first time in both lungs. At 10 p. m., hydrargyrum bichlorid., one one-hundredth grain, and ipecac, was begun—a dose every two hours. At night patient slept quietly and well, with scarcely any cough.

"At 7 a. m. of the 15th, somewhat hoarse; appetite better; bright and playful; looks pale and has lost considerable flesh; belly-ache is gone; bowels are normal; cough raw, dry, not hoarse, not severe; mercury and ipecac continued.

"On the 16th, the bronchial catarrh was in resolution; soft, mucous rales had taken the place of the dry subilant; cough loose, not hoarse: the boy was bright and playful; appetite had fully returned; bowels moved normally. The medication was kept up till the 17th, when there was no further occasion for its continuance.

### REMARKS.

"The change from 9 to 12 o'ctock ot the first day was something wonderful. Several hours before vomiting occurred respiration had become easy, and the obstruction in the larynx softened. Patient was lying in a peaceful slumber, the anxious expression of the morning entirely effaced.

"As further proof of the melting away of the exudation, we find a few hours later the voice partly resuming its timbre. Quite a change from the weak, dead, flat sound of the morning. The vomited matter evidently contained portions of membrane. The act of vomiting was a sudden and powerful effort. There was not much gagging, and after the stomach was relieved, quiet was restored. The vomiting was evidently caused by the mercury. There were but six easy feculent passages in the forty-eight hours of the mercurial treatment. There was no straining, but considerable tormina set in on the second day, wbich at once disappeared when the dose of the mercurial was diminished. The bronchitis following upon the croup was evidently equally benefitted with the primary disease. The mental hebetude, feverishness, extremely rapid, soft and small pulse, pale, bloated face, swollen neck, steady increase in the laryngeal symptoms notwithstanding the previous treatment, justify the diagnosis of true croup; and this opinion is confirmed by the flakes of membrane vomited and the course of the disease, corresponding, as it does, in all respects with that described by Dr. Reiter, the originator of this method of treatment.'" —*Medical and Surgical Reporter.*

---

An Indiana boy, only ten years of age, has a peculiar affliction. One of his ears is over a foot in diameter. It mortifies him terribly, as folks who see him on that side invariably mistake him for a Milwaukee editor.

